# Mast cell density in gastric biopsies of pediatric age group and its relation to inflammation and presence of Helicobacter pylori

**DOI:** 10.1186/1746-1596-2-14

**Published:** 2007-05-12

**Authors:** Fatemeh E Mahjoub, Behnam Hassanbeglou, Zahra Pourpak, Fatemeh Farahmand, Nasim Kashef, Atousa Azam Akhlaghi

**Affiliations:** 1 Pathology Department, Children Medical Center, Tehran University of Medical Sciences (TUMS), Tehran, Iran; 2 Immunology, Asthma and Allergy Research Institute, Children Medical Center, Tehran University of Medical Sciences (TUMS), Tehran, Iran; 3 Gastroenterology Department, Children Medical Center, Tehran University of Medical Sciences (TUMS), Tehran, Iran; 4 Nutrition Department, Children Medical Center, Tehran University of Medical Sciences (TUMS), Tehran, Iran

## Abstract

**Background:**

The aim of this study was to investigate the relationship between mast cell density, histological severity of gastritis, and presence of Helicobacter pylori (H. pylori) in the antral mucosa of pediatric patients.

**Methods:**

The study included 352 (192 male and 160 female, < 14 years old) patients. All cases underwent endoscopy, and biopsies were obtained for histopathological examination and evaluation of Helicobacter pylori. All biopsies were evaluated according to the Sydney system and mast cell density in the antral mucosa was analyzed by Giemsa stain. Spearman's correlation test was used to determine the relationship between mast cell density and other histopathological parameters. The comparison of mast cell density between H. pylori positive and negative groups was analyzed by Mann Whitney U test.

**Results:**

Mast cell density was 12.6 ± 0.87 in 0.25 mm^2 ^(0–81). Means of severity of gastric inflammation in H. pylori-positive and negative patients were 1.7 ± 0.6 and 0.6 ± 0.7, respectively, which was statistically significant (p = 0.0001). Mast cell density was not correlated with presence and degree of inflammation, activity, presence and score of H. pylori in the antrum (p > 0.05). There was no significant correlation between mast cell density and allergy.

**Discussion:**

We concluded that there may be some other ways for contribution of mast cells in pathologic processes involving gastrointestinal tract in children.

## Background

Mast cells are round to oval cells of connective tissue, measuring 20–30 micrometer in diameter with cytoplasmic granules containing glycosaminoglycans resulting in metachromasia and also histamine, neutral proteases, platelet activating factor and many others [1]. The surface of mast cells contains specific receptors for IgE, which after fixation and binding with special antigens, can result in release of mast cell granules and hence immediate hypersensitivity reaction [1].

Mast cells originate from bone marrow and are of the most important cells in inflammatory processes. While they release several mediators and cytokines at the beginning of inflammatory process, they are regarded as proinflammatory cells by some authors [2]. They have important role in triggering and regulating inflammatory processes. They reside adjacent blood and lymphatic channels mainly under epithelial surfaces such as skin, respiratory, gastrointestinal and urogenital tract [3].

Since 1996 few studies have been performed on mast cell density in gastrointestinal biopsies, mainly in adult age group. Nakajima et al. demonstrated the presence of mast cells by immunohistochemical staining with anti human tryptase antibody in gastric biopsies and concluded that density of mast cells was 2 to 3 times greater in H. pylori infected gastric mucosa than in negative normal stomach [4]. Bamba et al. demonstrated that increase in gastric mast cells in H. pylori positive biopsies is due to in situ proliferation of mast cells rather than migration from other sites and results from stem cell factor and Interleukins 3, 4 and 6 [5]. Sulik et al. studied mast cell involvement in children with or without infection by H. pylori. Results of this study showed that mast cell through its numerous mediators may play a key role in chronic gastritis especially in H. pylori positive cases [6]. Mysorekar et al. investigated the extent of mast cell involvement in antral gastritis with and without H. pylori infection in subjects with symptoms suggestive of acid peptic disease. They concluded that H. pylori could be responsible for increasing the mast cell density in the gastric antrum [7]. Kayaselcuk et al. investigated the relationship between mast cell density, H. pylori density and histopathological severity of gastritis in the corpus and antral mucosa and concluded that mast cell density was significantly higher in H. pylori positive group than negative group and also the higher mast cell distribution was correlated with increased inflammation and activity [8]. Maciorkowska et al. evaluated biopsy specimens of gastric mucosa collected from H. pylori-positive patients, patients after H. pylori infection and H. pylori-negative children. The specimens were assessed for infection and inflammation and stained with anti-human mast cell tryptase to count mucosal mast cells. In morphometric evaluation, slight differences were found in the numbers of mast cells among groups which was not statistically significant (the number of mastocytes being: 86.4, 81.4 and 70.2 cells/mm2 of specimen, respectively) [9].

As mentioned above, studies on mast cell density have been performed mainly on adults and on rather small groups as yet. So we decided to study mast cell density in pediatric age group on rather larger number of cases in a referral children hospital with longstanding experience in pediatric gastroenterology.

The aim of this study was to investigate the relationship between mast cell density, histopathological findings and presence of Helicobacter pylori in the antral mucosa in pediatric patients.

## Methods

352 children (< 14 years old, with gastrointestinal complaints) who referred to Children Medical Center, Tehran University of Medical Sciences with different gastrointestinal problems were enrolled in this study from 2004–2006, by sequential sampling. All cases underwent endoscopy, and antral biopsies were obtained for histological examination and evaluation of Helicobacter pylori. A questionnaire was filled for each patient including clinical and endoscopic findings and history of food and other allergies.

The specimens were fixed in 10% buffered formalin, processed, embedded in paraffin and cut in sequential 3 micrometer sections. Superficial and deep sections were stained by haematoxyline-eosin (two slides) and one slide was stained by Giemsa stain. Gastric severity was determined by Sydney scoring system and presence and scoring of H. pylori were determined by criteria in table 1.

**Table 1:** Modified scoring system for H. pylori density.

**Density of H. pylori**	**Histological features**
0	No Bacteria
1	Few bacteria (5 to 10) observed only under ×40 magnification
2	Patchy distribution (<50 of total surface crypts) with lower density (NCB<10)
3	Patchy distribution (<50 of total surface crypts) with higher-density (NCB>10) Or diffuse distribution (>50 of total surface crypts) with mixture of low-density (NCB<10)
4	Diffuse distribution (>20 of total surface crypts) with mixture of high (NCB>10) and low-density (NCB<10)
5	Diffuse distribution (>50 of total surface crypts) all with high density (NCB>10)

NCB = Number of H. pylori in each crypts

Mast cells were counted by Giemsa stain at ×1000 magnification in 10 fields with a Zeiss standard 20 light microscope and the sum was calculated for each case (measuring 0.25 mm^2^). All the mast cell counts and histological evaluation was performed by a single observer.

The statistical analysis was performed using SPSS, version 11.5 (SPSS Inc., Chicago, IL, USA). Values were tested for normality using a Kolmogorov-Smirnov test. Spearman's correlation test was used to determine the relationship between mast cell density and other histopathological parameters. The comparison of mast cell density between H. pylori positive and negative groups was analyzed by Mann Whitney U test. A P-value of 0.05 or less was considered significant.

## Results

Over a 2 year period of study, 352 antral biopsies were obtained from participants. Among these persons, 54.5% (192 persons) were male. There was no significant difference between sex of patients (p > 0.05). The mean age of patients included in the study was 7.17 ± 3.27 (range: 1–14 years). Table 2 provides an indication of patients' baseline characteristics and clinical diagnosis. As shown in table 2, 119 persons (33.8%) of patients had pain before eating, 102 persons (28.9%) pain after eating, 49 persons (13.9%) weight loss, and 47 persons (13.5%) had heart burn. No significant relation was found between sex and type of gastrointestinal complaint. 69 persons (19.6%) were positive for allergy history. Endoscopic findings are depicted in table 2.

**Table 2:** Patients' baseline characteristics and clinical diagnosis based on endoscopic findings.

	**male (n = 192)**	**female (n = 160)**	**p-value**
Age (yr)*	7 ± 3.5	7.3 ± 3.1	NS^a^
**Symptoms (%)**			
Pain before eating	66 (18.7%)	53 (15.1%)	NS^b^
Pain after eating	60 (17%)	42 (11.9%)	NS
Weight loss	29 (8.2%)	20 (5.7%)	NS
Heart burn	23 (6.6%)	24 (6.8%)	NS
Others	55 (15.6%)	38 (10.7%)	NS
**History of allergy (%)**	34(17.7%)	35 (21.8%)	NS
**Clinical diagnosis (%)**			
Based on Endoscopic Results			
Normal	85 (24.1%)	75 (21.3%)	NS
Esophagitis	32 (9.1%)	25 (7.1%)	
Gastritis	37 (10.5%)	30 (8.5%)	
Duodenitis	3 (0.9%)	3 (0.9%)	
Gastroesophagitis	35 (9.9%)	27 (7.7%)	

Data is shown as mean ± SD.

^a^Not significant (NS), student's t test.

^b^chi square test

As shown in table 3, of 352 cases, 36.6% of gastric biopsies showed no significant pathologic change, 29% mild chronic gastritis, 10.2% moderate chronic gastritis, 1.1% severe chronic gastritis, and 23% follicular gastritis. 15.6% also showed activity. 17.6% of cases were positive for H. pylori. Means of severity of gastric inflammation in H. pylori-positive and negative patients were 1.7 ± 0.6 and 0.6 ± 0.7, respectively, which was statistically significant (p = 0.0001). There was a statistically significant relation between H. pylori status and the activity of gastritis (p = 0.0001).

**Table 3:** Pathologic characteristics

	**male (n = 192)**	**female (n = 160)**	**p-value**
**Gastritis (%)**			
Normal	70 (19.9%)	59 (16.8%)	0.166
Mild	55 (15.6%)	47 (13.4%)	
Moderate	26 (7.4%)	10 (2.8%)	
Severe	1 (0.3%)	3 (0.9%)	
Follicular	40 (11.4%)	41 (11.6%)	
**Helicobacter pylori (%)**	34 (9.7%)	28 (8%)	0.537
**Activity (%)**	30 (8.5%)	25 (7.1%)	0.560
**Mast cell density**			
mean (range)	13.1 (0–78)	12.2 (0–81)	NS
Median	7	5	

^a ^Not significant (NS)

**Figure 1:**
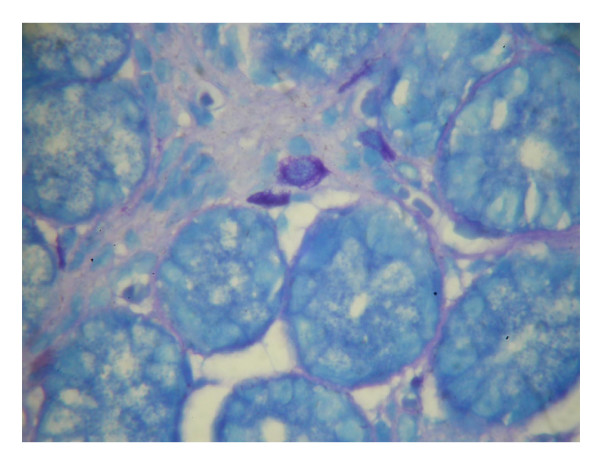
Mast cells seen by Giemsa staining as large oval cells containing metachromatic granules (five are seen in this field, oil immersion ×1000, Giemsa stain).

**Figure 2:**
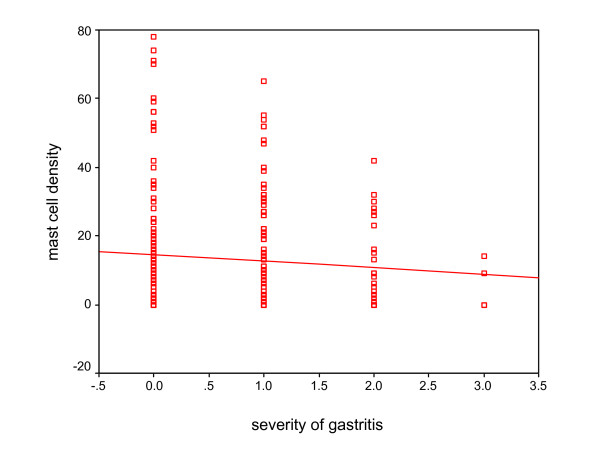
The relationship between mast cell density and severity of gastritis (r = -0.032, p = 0.604).

**Figure 3:**
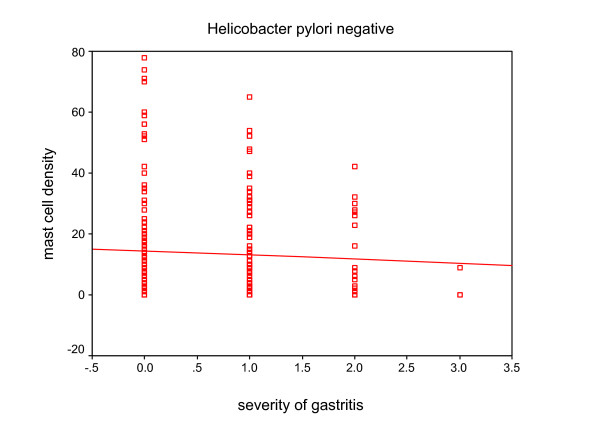
The relationship between mast cell density and severity of gastritis in H. pylori negative patients (r = -0.003, p = 0.962).

**Figure 4:**
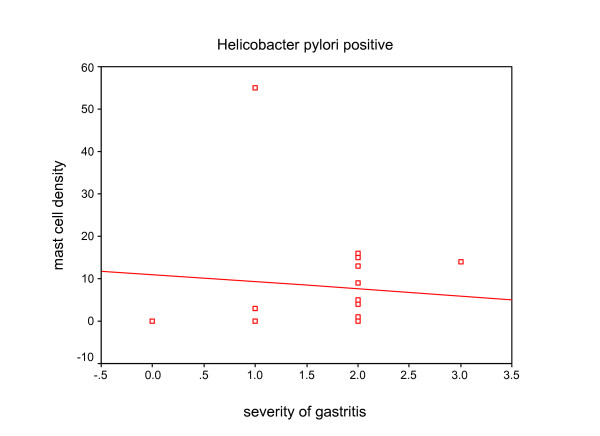
The relationship between mast cell density and severity of gastritis in H. pylori positive patients (r = 0.182, p = 0.485).

Mast cell density was 12.6 ± 0.87 in 0.25 mm^2 ^(range: 0–81) (Fig. 1). Mast cell density, presence and degree of inflammation based on H. pylori status is presented in table 4. We found no significant correlation between mast cell density and sex of patients. Also no significant correlation was found between mast cell density and presence and degree of inflammation [(r = -0.032, p = 0.604), (Fig. 2)], activity (p = 0.09) and presence of H. pylori (p = 0.21). According to H. pylori status, there was no statistically correlation between mast cell density and severity of gastritis (Fig. 3 and 4). No significant difference was found between mast cell density and history of allergy (p = 0.34).

**Table 4:** Mast cell density and presence and degree of inflammation.

**gastritis**	**mast cell density**		Total (n = 352)
		
	HP (+) (n = 62)	HP (-) (n = 290)	
normal	0	14 ± 18.6 (6)	13.9 ± 18.5 (5.5)
mild	19.3 ± 30.9 (3)	13.7 ± 15.1 (9)	9.4 ± 11.8 (4.5)
moderate	5.2 ± 6.3 (2.5)	11.7 ± 13.5 (5.5)	9.4 ± 11.8 (4.5)
severe	14	3 ± 5.1 (0)	5.7 ± 6.9 (4.5)
follicular	10.1 ± 14.5 (5)	12.1 ± 15 (4)	11 ± 14.6 (5)
total	9.5 ± 14.1 (4)*	13.3 ± 16.5 (6.5)	

Data is shown as mean ± SD (median)

HP: Helicobacter pylori

*Mann-Whitney test; not statistically significant (p = 0.217)

## Discussion

Mast cells are claimed to be an important element in the cellular infiltration in the course of gastric mucosal inflammation due to H. pylori infection [10]. There is only little data in literature concerning the number of mast cells in chronic inflammation of the gastric mucosa in pediatric age group and existing data have rather conflicting results.

Mast cell detection is rather easy by a simple and inexpensive staining (Giemsa) which can also be used to detect presence of H. pylori. It is of worth noting that reliable detection is achieved by examining the tissue under oil immersion (x1000) and in lower magnifications they can be missed. Although the gold standard is staining of tissue with anti-tryptase antibody by immunohistochemical methods, it is a time consuming and rather expensive method not recommended for routine pathologic assessment of gastric biopsies.

We decided to study on rather large number of specimens in a referral children hospital with longstanding experience in pediatric gastroenterology and determine mast cell density and its relation to inflammation and presence of H. pylori.

In this study, we did not observe a significant correlation between mast cell density, severity of inflammation, activity of gastritis and presence and density of H. pylori in antrum. Our results are rather in concordance with results of few authors such as Maciorkowska et al., but not others [6-8], although none of these studies were performed on such large series as ours.

H. pylori is the most frequent and significant factor in the etiology of chronic active gastritis, in addition to being implicated in various illnesses such as peptic ulcer, gastric adenocarcinoma, and lymphoma [8]. Our results also showed that there was a significant relationship between infection with H. pylori and severity of gastritis (p = 0.0001).

Mast cells are the most important cells in acute allergic reactions with mediation of IgE. In this study, there was no significant correlation between allergic symptoms stated by parents (food and other allergies) and gastric mast cell density.

Many children undergo endoscopy with common complaint of chronic or recurrent abdominal pain in our center. They are mainly in range of 5 to 7 years of age and no significant changes are observed both endoscopically and by microscopic examination, as in many of our cases (nearly 231 out of 352 cases). Our study shows that mast cell density in this group is slightly higher than other groups (although statistically not significant) and we propose that maybe mast cells play a role in producing such symptoms not by their traditional known pathways in inflammatory processes but by an as yet undefined pathway such as stimulatory effect on gastric nerve plexuses. So we recommend that increase in mast cell density should be included in the final report of gastric biopsies.

Although we did not performed immunohistochemical stains for mast cell detection, we propose that Giemsa stain is a reliable and easy staining method available in all laboratories and also has good interobserver correlation. Although it has also shortcomings such as erroneous detection of artifactual staining as mast cells, effects of edema and shrinkage and also missing them because of degranulation, these have no significant impact on the final count.

Besides the shortcomings of our study it is necessary to mention that it was performed on rather a large series of pediatric patients and the results are not surprisingly concordant with other studies mainly performed in adult age group.
